# Physiological Evaluation of Alkali-Salt Tolerance of Thirty Switchgrass (*Panicum virgatum*) Lines

**DOI:** 10.1371/journal.pone.0125305

**Published:** 2015-07-06

**Authors:** Guofu Hu, Yiming Liu, Xunzhong Zhang, Fengjiao Yao, Yan Huang, Erik H. Ervin, Bingyu Zhao

**Affiliations:** 1 College of Animal Science and Technology, Northeast Agricultural University, Harbin, Heilongjiang Province, P.R. China; 2 Department of Crop and Soil Environmental Science, Virginia Polytechnic Institute and State University, Blacksburg, Virginia, United States of America; 3 Department of Horticulture, Virginia Polytechnic Institute and State University, Blacksburg, Virginia, United States of America; Chinese Academy of Sciences, CHINA

## Abstract

Soil salt-alkalization is a major limiting factor for crop production in many regions. Switchgrass (*Panicum virgatum* L.) is a warm-season C_4_ perennial rhizomatous bunchgrass and a target lignocellulosic biofuel species. The objective of this study was to evaluate relative alkali-salt tolerance among 30 switchgrass lines. Tillers of each switchgrass line were transplanted into pots filled with fine sand. Two months after transplanting, plants at E5 developmental stage were grown in either half strength Hoagland’s nutrient solution with 0 mM Na^+^ (control) or half strength Hoagland’s nutrient solution with 150 mM Na^+ ^and pH of 9.5 (alkali-salt stress treatment) for 20 d. Alkali-salt stress damaged cell membranes [higher electrolyte leakage (EL) ], reduced leaf relative water content (RWC), net photosynthetic rate (Pn), stomatal conductance (g_s_), and transpiration rate (Tr). An alkali-salt stress tolerance trait index (ASTTI) for each parameter was calculated based on the ratio of the value under alkali-salt stress and the value under non-stress conditions for each parameter of each line. Relative alkali-salt tolerance was determined based on principal components analysis and cluster analysis of the physiological parameters and their ASTTI values. Significant differences in alkali-salt stress tolerance were found among the 30 lines. Lowland lines TEM-SEC, Alamo, TEM-SLC and Kanlow were classified as alkali-salt tolerant. In contrast, three lowland lines (AM-314/MS-155, BN-13645-64) and two upland lines (Caddo and Blackwell-1) were classified as alkali-salt sensitive. The results suggest wide variations exist in alkali-salt stress tolerance among the 30 switchgrass lines. The approach of using a combination of principal components and cluster analysis of the physiological parameters and related ASTTI is feasible for evaluating alkali-salt tolerance in switchgrass.

## Introduction

Salt-alkalization is a major global environmental and land resource issue. There is approximately 0.25 to 0.5 Mha of agricultural land lost annually due to soil salinization worldwide, especially in arid and semiarid areas [1]. Soil salinization and alkalization, frequently occurring together, significantly reduce crop productivity [2–4]. It is estimated that there are 950 million ha of saline-alkalized land worldwide [5]. Given that the cultivated area of alkaline soils (37% = 0.56 x 10^9^ ha) is larger than that of saline soils (23% = 0.34 x 10^9^ ha) [6], soil alkalinity constrains crop production more than soil salinity.

Breeding for alkali-salt tolerant plant species and cultivating these species on alkali-salt soil has been an effective approach for alleviating soil salinization and improving saline-alkalized soil. Establishing tolerant plant species on saline-alkalized soil can not only alleviate soil salinization but also achieve economic return. Switchgrass has been considered as one of the ideal plant species to alleviate the salinization of soil. Switchgrass is a warm-season C_4_ perennial steppe plant which is native to North America (Great Plains and much of the eastern part). It distributes naturally from 55°N latitude in Canada into the United States and Mexico [7–9]. Historically, this species has been classified into two main ecotypes based on its morphology and habitat: upland and lowland [10]. Because switchgrass requires relatively modest levels of fertilizers [11–14], it can grow on marginal lands, including millions of hectares of salinized lands, to avoid competition with arable lands. This grass can be used for soil conservation because of its strong root system [15]. Switchgrass is not only used as a forage with high biomass potential [11, 12], but also for large scale lignocellulosic biofuel production [16, 17]. It can survive for ten years or longer once it is established via seed and/or rhizome propagation. Selection of switchgrass lines with improved tolerance to alkali-salt stress has been one of the major goals of switchgrass breeding programs.

High pH in alkali-salt soil may significantly affect photosynthesis and photosynthetic electron transport [18]. Yang et al. [19] found that the alkali-salt induced decline in Pn was related not only to destruction of photosynthetic pigments and decreasing of g_s_, but also to imbalances of intracellular Na^+^ and K^+^. The high pH caused by alkali-salt may damage photosynthetic activity. Moreover, the high-pH environment surrounding the roots may directly cause Ca^2+^, Mg^2+^ and H_2_PO_4_^-^ to precipitate [20], inhibit ion uptake [19], and disrupt the ion homeostasis of plant cells. The level of cell membrane damage can be measured by an increase in electrolyte leakage (EL). Photosynthetic efficiency can be determined by measuring leaf net photosynthetic rate (Pn), stomatal conductance (g_s_), and transpiration rate (Tr) [21–28]. Leaf relative water content (RWC) is also a commonly used index for evaluating alkali-salt stress tolerance. Previous reports indicate that RWC decreases as intensity of saline-salt and alkali-salt stress increases. The extent of the decreases in RWC under alkali salt was greater than those observed under saline-salt [3, 29–33]. The physiological parameters (Pn, g_s_, Tr, EL and RWC) are closely associated with overall photosynthetic function, and could be used as markers for screening alkali-salt tolerance in various plant species.

Evaluating and identifying plant materials that are tolerant to alkali-salt stress is important for plant breeding programs. However, no research has been reported on evaluation of alkali-salt stress of switchgrass lines using the physiological parameters as described previously. Therefore, the objectives of this study were: 1) to determine the physiological responses of 30 lowland and upland switchgrass lines, 2) to identify alkali-salt tolerant and sensitive switchgrass lines, and 3) to establish a screening model for plant tolerance to alkali-salt stress by principal component and cluster analysis.

## Materials and Methods

### Plant Materials and Growth Conditions

This study was carried out in the greenhouse facility of Virginia Tech, Blacksburg, VA, USA from February through June, 2014. A total of 30 switchgrass lines were used in this study. Experimental lines included AM-314/MS-155, Alamo, TEM-SEC, Kanlow, TEM-LoDorm, BN-13645-64, TEM-SLC, 70SG0019, Pathfinder, Blackwell-3, BN-8624-67, Cave-in-Rock, 70SG0023, T16971, 70SG001, BN-12323-69, Caddo, 70SG002, BN-18758-67, 70SG0018, T-2086, Sunburst, BN-11357-63, Summer, Grenville-2, 70SG0022, BN-10860-61, Grif Nebraska 28, Blackwell-1 and Dacotah. Among the 30 lines, AM-314/MS-155, Alamo, TEM-SEC, Kanlow, TEM-LoDorm, BN-13645-64 and TEM-SLC were lowland ecotypes, while the rest were upland ecotypes.

The diverse switchgrass lines were originally obtained from the United States Department of Agriculture Germplasm Center and grown in field plots at the Virginia Tech research farm in Blacksburg, VA. Tillers of each line were collected from the field plots in September, 2013, and grown in pots in a greenhouse at Virginia Tech, Blacksburg, VA. About one dozen tiller buds with a similar size from each line were transplanted into plastic pots (15 cm diameter on the top, 10.5 cm diameter at the bottom, and 14 cm deep, with eight 10 mm drainage holes at the bottom) filled with sand on Jan. 21, 2014. A piece of plastic screen was placed in the bottom of the pot to prevent sand from leaching. The pots were kept in the greenhouse with temperature of 28±1°C during the day and 24±1°C during the night, photosynthetic active radiation (PAR) at 400 μmol ^-2^ s ^-1^, and a 14 h photoperiod. In addition, the seedlings were irrigated daily and fertilized twice a week with half-strength Hoagland’s solution [34].

When the plants reached E5 developmental stage [35], they were subjected to alkali-salt stress treatment. The alkali-salt treatment solution, which consisted of equal molarity of Na_2_CO_3_ and NaHCO_3_, with a concentration of Na^+^ at 150 mM and pH of 9.5, was prepared with half strength Hoagland’s nutrient solution. The solution for the control group was half strength Hoagland’s only. In order to avoid stress shock [36], the concentration of alkali-salt solution was applied at 50 mM on the first day (22 March, 2014) and then gradually increased to 150 mM at an increase of 50 mM each day. Then each pot was placed in a plastic tray (16 cm diameter, 6.5 cm deep) filled with either alkali-salt treatment solution or half strength Hoagland’s nutrient solution only. The lower third portions of the pots were submerged in the solutions all the time. Half strength Hoagland’s nutrient solution was added daily to maintain the same level of solution in the tray. After six days of treatment, samples were taken for analysis. The experiment was completed at day 20 (10 April, 2014).

### Measurements

All measurements took place at six days after initial treatment. Some lines started wilting after day 6 and were lost due to alkali-salt stress injury after day 10. Therefore, no physiological measurements were made after day 6.

#### Photosynthetic parameters

Pn, g_s_, and Tr were measured using a portable photosynthesis system (Li-6400 XT, LICOR, Inc, Lincoln, NE, USA) under controlled atmosphere conditions (385 μmol mol^−1^ CO_2_, 500 μmol s^-1^ flow rate, 26°C) and a Licor 6400 LED external light source (a photosynthetic photon flux density (PPFD) at 2000 μmol m^−2^ s^−1^). The uppermost fully expanded leaf on the main tiller in each pot was selected for measurements. Three readings were collected for each sample and the average was used for statistical analysis.

#### Leaf electrolyte leakage

Leaf EL was measured using Marcum’s method [37] with some modifications. The top 2nd-3rd mature leaf was excised and cut into 2 cm segments. After being rinsed three times with distilled de-ionized H_2_O, 0.2 g leaf segments were placed in a test tube containing 20 mL distilled de-ionized H_2_O. The test tubes were agitated on a shaker for about 24 h and the solution conductivity (C_1_) was measured with a conductivity meter (Symphony SR601C pH/EC meter, VWR Corporation, Radnor, PA). Leaf samples were then autoclaved at 100 ^o^C for 30 min, and the conductivity of the solution containing killed tissue was measured once the tubes cooled down to room temperature (C_2_). The relative EL was calculated as (C_1_/ C_2_) ×100.

#### Leaf relative water content

Leaf RWC was measured using Weatherley’s method [38] with some modifications. The top 2^nd^-3^rd^ mature leaf was excised and cut into small segments. Leaf segments, weighed up to 0.2 g fresh, were then immediately placed in de-ionized H_2_O in capped containers to maintain approximately 100% relative humidity in the container in order for leaves to reach full hydration at room temperature. It takes approximately overnight for leaves to be fully rehydrated. Samples were removed from water and lightly blotted dry to remove all surface moisture, and then weighed for turgid weight. Dry weight was then determined for samples dried in an oven at 75–85 ˚C for approximately 48–72 hours. The RWC % was calculated as (fresh weight–dry weight)/ (turgid weight–dry weight) ×100.

#### Alkali-salt tolerance trait index

The STTI (salt tolerance trait index) has been used to assess the salt tolerance of different lines in past studies. STTI was calculated using the formula: STTI = (value of trait under salt stress condition)/(value of trait under controlled condition) × 100 [39–41]. Based on the concept of STTI, ASTTI (Alkaline-salt tolerance trait index) was adopted in this study to estimate alkali-salt tolerance.

### Experimental Design and Statistical Analysis

When plants reached the E5 developmental stage, eight pots with uniform growth from each line were selected for the alkali-salt stress experiment. A split plot design was used with alkali-salt stress treatments as the main plots and lines as the subplots. The main plots consisted of alkali-salt stress treatment and control (no alkali-salt stress). The alkali-salt stress and control groups were physically paired with each other and randomly placed in each of four pairs (replicates). The subplots consisted of 30 lines. The 30 lines in each main plot were randomly placed in each replicate. There were four replications. All data were subjected to analysis of variance. Mean separations were performed using Fisher's protected least significant difference (LSD) test at 5% level.

Correlation, principal component analysis, and cluster analysis were carried out for all traits. Synthesis scores derived from principal component analysis were used to determine relative alkali-salt tolerance among the 30 lines [42]. Eigenvectors (Y_1_) were calculated based on the following formula:

$$Y_{1}=-0.324EL\;+\;0.043RWC\;+\;0.542Pn\;+\;0.546g_{s}+\;0.550Tr$$ (1)

The coefficient for each parameter in formula (1) was from the related A_1_ value in Table 1. Similarly, the Y_2_ and Y_3_ were calculated based on the relative values of A_2_ and A_3_ in Table 1. The Y_1_, Y_2_, and Y_3_ values of each line were calculated based on the ASTTI value of each physiological parameter for the given line and listed in Table 2. The synthesis score for each line was calculated by the formula (2) using Eigenvalues (λ) as a weighted index and are listed in the right column in Table 2.

$$Y=\frac{\lambda_{1}}{\sum_{i=1}^{3}\lambda_{i}}\;Y_{1}+\frac{\lambda_{2}}{\sum_{i=1}^{3}\lambda_{i}}\;Y_{2}+\frac{\lambda_{3}}{\sum_{i=1}^{3}\lambda_{i}}\;Y_{3}$$ (2)

Where *λ*_1_ = 2.994, *λ*_2_ = 1.127, *λ*_3_ = 0.654. $\sum_{i-1}^{3}\lambda_{i}=\lambda_{1}+\lambda_{2}+\lambda_{3}=2.994+1.127+0.654=4.075$ (Table 2)

All statistical analyses and calculations were conducted using SAS v. 9.3 software [43].

**Table 1:** Principal component analysis among physiological measurements in 30 switchgrass lines under alkali-salt stress conditions.

Eigenvector	λ_1_	λ_2_	λ_3_
	2.994	1.127	0.654
Cumulative	0.599	0.824	0.955
Physiological		Eigenvectors	
Parameters	A_1_	A_2_	A_3_
EL	-0.324	-0.474	0.813
RWC	0.043	0.855	0.509
Pn	0.542	-0.139	0.223
g_s_	0.546	-0.141	0.055
Tr	0.550	-0.069	0.165

**Table 2:** Eigenvectors and synthesis scores of the physiological parameters in 30 switchgrass lines under alkali-salt stress conditions.

Population	Eigenvectors			
	Y_1_	Y_2_	Y_3_	Y (Synthesis score)
TEM-SEC	0.673	-0.115	2.020	0.670
Alamo	0.656	-0.063	1.971	0.665
TEM-SLC	0.589	-0.169	2.027	0.605
Kanlow	0.569	-0.110	2.014	0.605
70SG001	0.308	-0.104	1.937	0.432
T16971	0.171	-0.083	2.067	0.369
70SG0023	0.233	-0.244	2.037	0.366
Grif Nebraska 28	0.163	-0.174	2.130	0.351
T-2086	0.118	-0.108	2.018	0.323
Grenville-2	-0.001	0.027	1.858	0.258
BN-10860-61	-0.073	0.006	1.903	0.214
Cave-in-Rock	-0.096	-0.003	1.918	0.200
TEM-LoDorm	-0.131	-0.028	1.988	0.182
BN-12323-69	-0.120	-0.178	2.068	0.164
Pathfinder	-0.155	-0.048	1.941	0.155
BN-18758-67	-0.034	-0.288	1.787	0.154
Dacotah	-0.166	-0.340	2.445	0.148
BN-11357-63	-0.182	-0.069	2.032	0.146
70SG002	-0.105	-0.318	2.064	0.140
70SG0018	-0.165	-0.047	1.870	0.140
Sunburst	-0.250	-0.114	2.136	0.107
Blackwell-3	-0.260	0.048	1.859	0.101
Summer	-0.285	-0.242	2.349	0.084
70SG0022	-0.296	-0.295	2.396	0.071
Blackwell-1	-0.335	-0.436	2.638	0.046
70SG0019	-0.437	-0.055	1.999	-0.015
BN-13645-64	-0.387	-0.736	2.886	-0.024
BN-8624-67	-0.447	-0.129	2.084	-0.027
Caddo	-0.463	-0.430	2.596	-0.039
AM-314/MS-155	-0.536	-0.670	2.749	-0.120

## Results

The two factors (alkali-salt stress treatment and line) and the interactions between them were statistically significant (P ≤ 0.05) for EL, RWC, Pn, g_s_, and Tr. Statistically significant differences in the physiological parameters were observed among the 30 switchgrass lines. The alkali-salt tolerant and sensitive lines were clearly identified based on the physiological parameters by cluster analysis and principal component analysis.

### Effects of Alkali-salt Stress on Leaf EL

**Fig 1:**
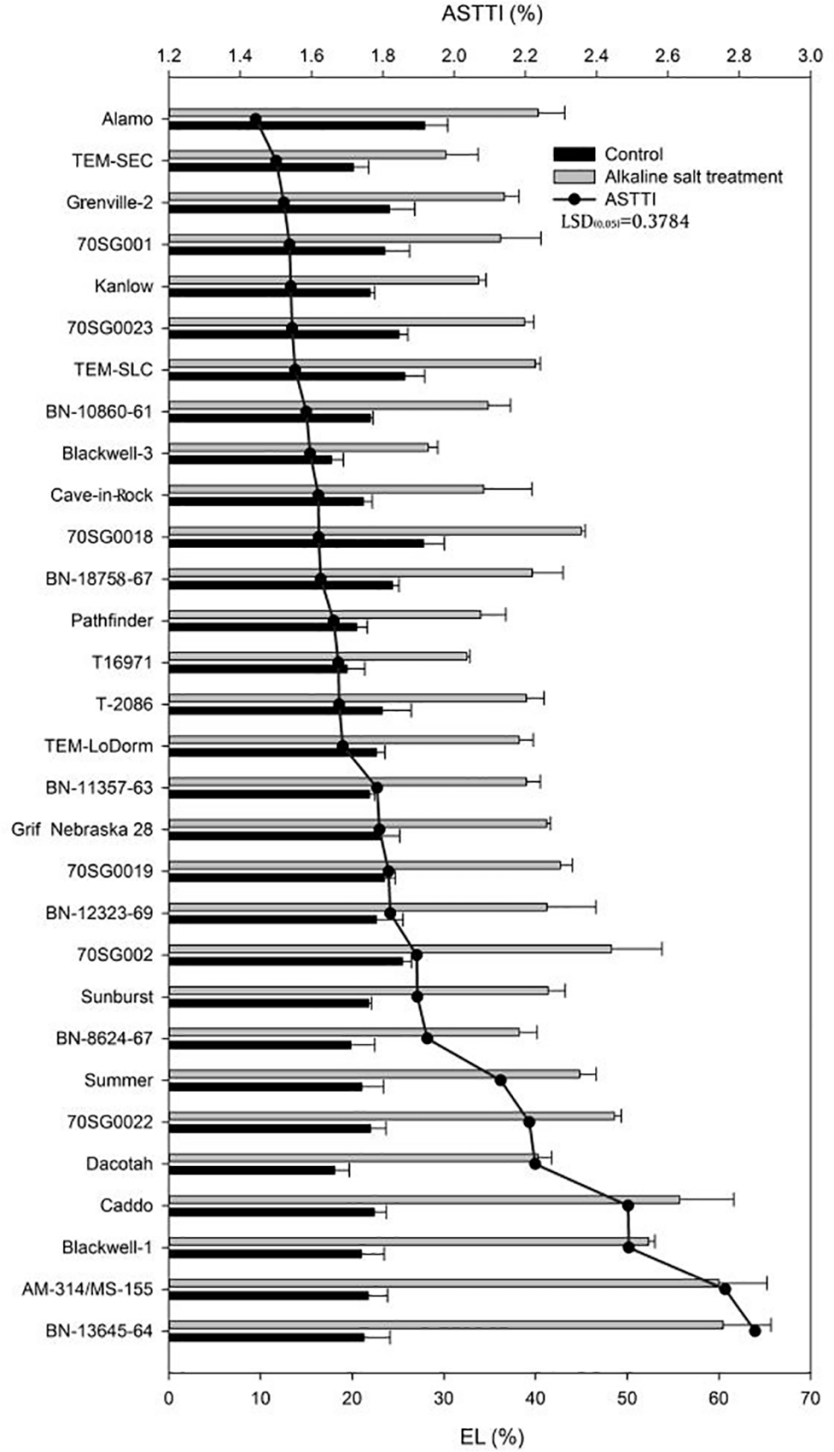
Leaf electrolyte leakage (EL) of 30 lines under control (solid bar) and alkali-salt stress (150 mM Na^+^ with pH of 9.5; open bar) at six days of alkali salt treatment. The line represents alkali-salt tolerance trait index (ASTTI). The lower the ASTTI, the better the alkali-salt tolerance. Values are means ±SEM (n = 4). The bar represents LSD (0.05) for ASTTI.

Leaf EL increased in response to alkali-salt stress in all lines (Fig 1). EL responses to alkali-salt stress varied greatly among the 30 lines, with Alamo, TEM-SEC, Grenville-2, 70SG001, Kanlow, 70SG0023 and TEM-SLC having less EL under alkali-salt stress, and ASTTI values less than 155%. Alamo had the least EL under alkali-salt stress, with an ASTTI at 144%. On the other hand, BN-13645-64, AM-314/MS-155, Blackwell-1, Caddo, Dacotah, 70SG0022, and Summer had greater EL increase under alkali-salt stress, with ASTTI values greater than 213%. BN-13645-64 had the highest EL, with an ASTTI value of 284%.

### Effects of Alkali-salt Stress on Leaf RWC

**Fig 2:**
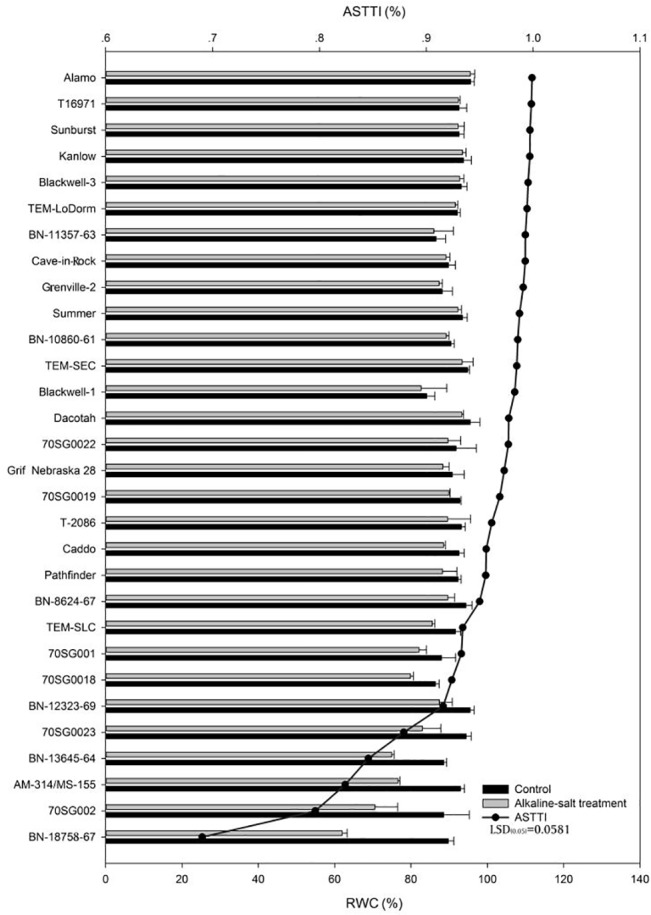
Relative water content (RWC) of 30 lines under control (solid bar) and alkali-salt stress (150 mM Na^+^ with pH of 9.5; open bar) at six days of alkali salt treatment. The line represents alkali salt tolerance trait index (ASTTI). The greater the ASTTI, the better the alkali salt tolerance. Values are means ±SE (n = 4). The bar represents LSD (0.05) for ASTTI.

Alkali-salt stress reduced leaf RWC in all lines (Fig 2). The RWC response to alkali-salt stress varied among lines. Alkali-salt induced decline in RWC was relatively small for Alamo, T16971, Sunburst, Kanlow, Blackwell-3, TEM-LoDorm, and BN-11357-63, with ASTTI values for these lines being greater than 99%. Alamo had the least reduction in RWC under alkali-salt stress, with an ASTTI of 100%. In contrast, Alkali-salt stress caused greater RWC reduction for BN-18758-67, 70SG002, AM-314/MS-155, BN-13645-64, 70SG0023, BN-12323-69, and 70SG0018, with ASTTI values being less than 92%. Among the lines, BN-18758-67 had the greatest reduction in RWC due to alkali-salt stress with an ASTTI value of 69%.

### Effects of Alkali-salt Stress on Pn

**Fig 3:**
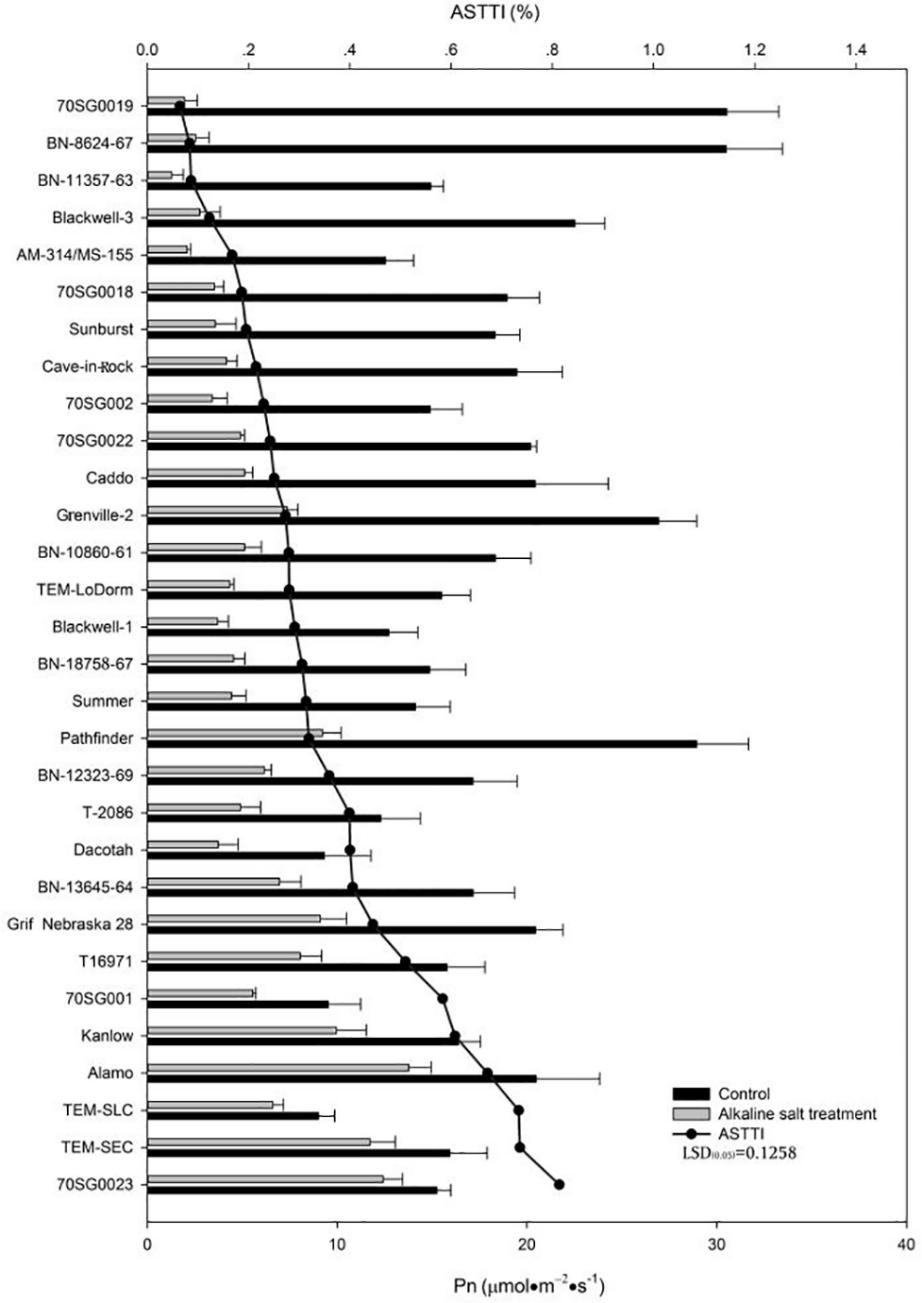
Photosynthetic rate (Pn) of 30 lines under control (solid bar) and alkali-salt stress (150 mM Na^+^ with pH of 9.5; open bar) at six days of alkali-salt treatment. The line represents alkali-salt tolerance trait index (ASTTI). The greater the ASTTI, the better the alkali salt tolerance. Values are means ±SE (n = 4). The bar represents LSD (0.05) for ASTTI.

Alkali-salt stress caused a reduction in Pn regardless of lines (Fig 3). Variations in Pn in responses to alkali-salt stress were found between lines. T16971, 70SG001, Kanlow, Alamo, TEM-SLC, TEM-SEC and 70SG0023 had less Pn decline, with ASTTI values greater than 51.0%. Among the lines, T16971 had the least Pn reduction due to alkali-salt stress, with an ASTTI of 81.4%. In contrast, 70SG0019, BN-8624-67, BN-11357-63, Blackwell-3, AM-314/MS-155, 70SG0018, and Sunburst had greater Pn reduction due to alkali-salt stress, with ASTTI values being less than 19.5%. The Pn decreased the most for BN-18758-67 and this line had an ASTTI value of 6.4%.

### Effects of Alkali-salt Stress on g_s_

**Fig 4:**
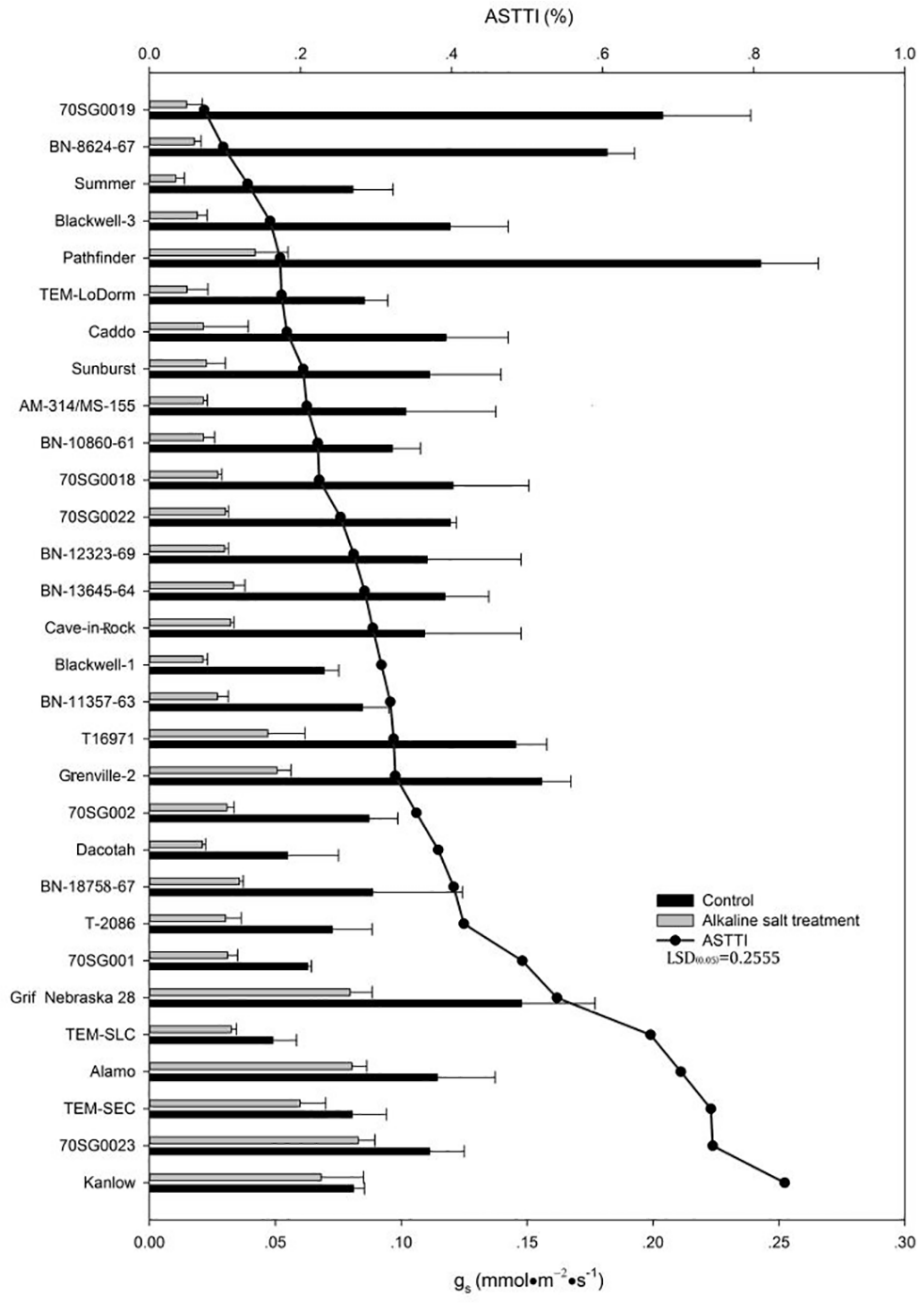
Stomatal conductance (g_s_) of 30 lines under control (solid bar) and alkali-salt stress (150 mM Na^+^ with pH of 9.5; open bar) at six days of alkali salt treatment. The line represents alkali-salt tolerance trait index (ASTTI). The greater the ASTTI, the better alkali salt tolerance. Values are means ±SE (n = 4). The bar represents LSD (0.05) for ASTTI.

Alkali-salt stress reduced g_s_ in all lines (Fig 4). The change of g_s_ in response to alkali-salt stress varied greatly among the 30 lines. 70SG001, Grif Nebraska 28, TEM-SLC, Alamo, TEM-SEC, 70SG0023, and Kanlow had less g_s_ reduction due to alkali-salt stress, with ASTTI values greater than 49.4%. 70SG001 had the greatest ASTTI of 84.0%. On the other hand, alkali-salt stress caused larger reductions in g_s_ for 70SG0019, BN-8624-67, Summer, Blackwell-3, Pathfinder, TEM-LoDorm, and Caddo, which had ASTTI values less than 18.2%. 70SG0019 had the lowest g_s_, with an ASTTI value of 7.3%.

### Effects of Alkali-salt Stress on Tr

**Fig 5:**
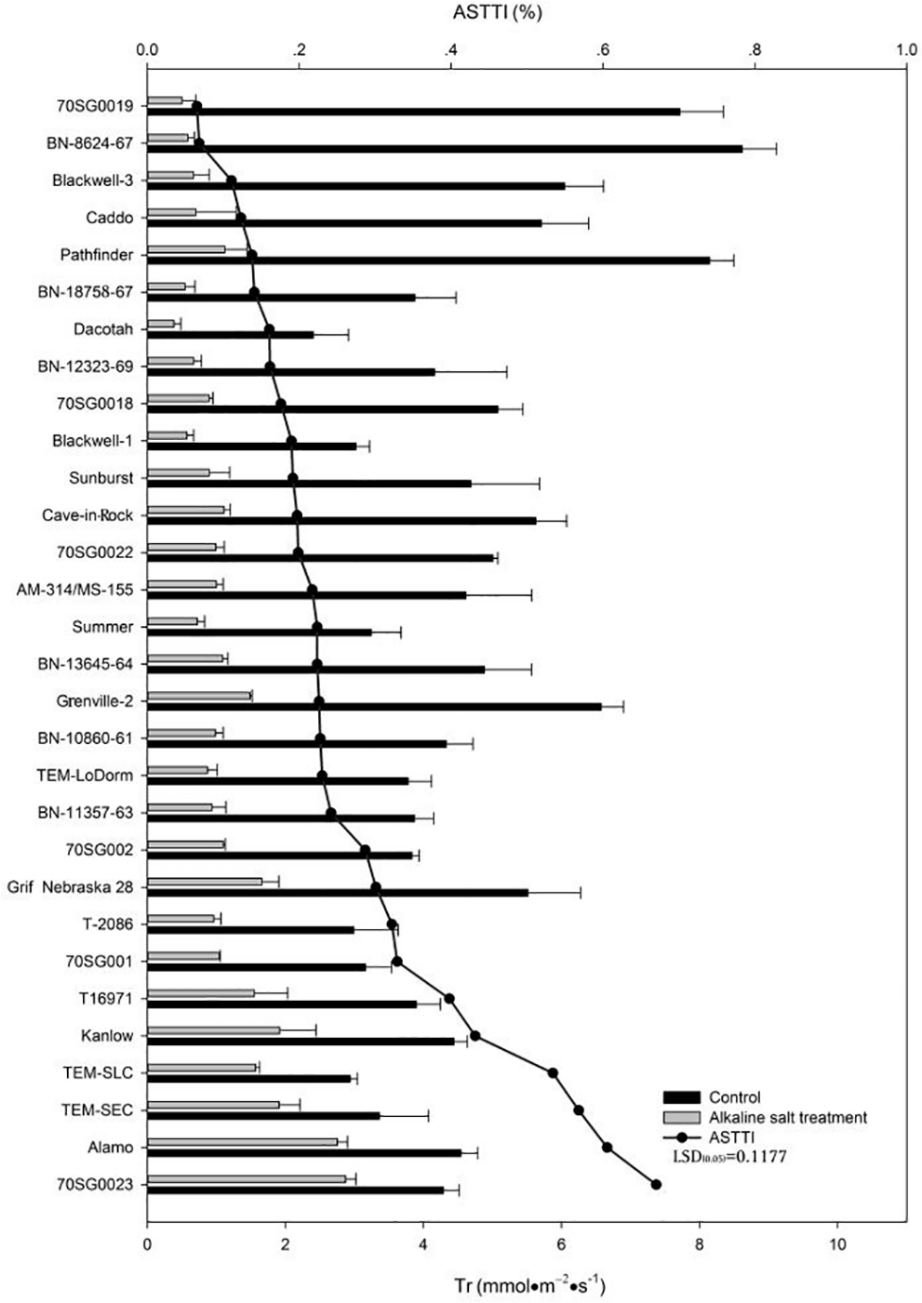
Transpiration rate (Tr) of 30 lines under control (solid bar) and alkali-salt stress (150 mM Na^+^ with pH of 9.5; open bar) at 6 days of alkali-salt treatment. The line represents alkali-salt tolerance trait index (ASTTI). The greater the ASTTI, the better the alkali salt tolerance. Values are means ±SE (n = 4). The bar represents LSD (0.05) for ASTTI.

Alkali-salt stress reduced Tr in all lines (Fig 5). Tr responses to alkali-salt stress varied among the lines. 70SG001, T16971, Kanlow, TEM-SLC, TEM-SEC, Alamo, and 70SG0023 had less Tr reduction due to alkali-salt stress, with ASTTI values greater than 32.9%. 70SG0023 had the least Tr reduction, with an ASTTI of 67.0%. On the other hand, 70SG0019, BN-8624-67, Blackwell-3, Caddo, Pathfinder, BN-18757-67, and Dacotah had greater Tr reduction due to alkali-salt stress, with ASTTI values less than 16.1%. Alkali-salt stress reduced Tr the most for 70SG0019 which had an ASTTI value of 6.5%.

### Correlation, Principal Component Analysis, and Clustering Analysis of Physiological Parameters

Correlations between the physiological parameters under alkali-salt stress conditions are shown in Table 3. There were negative correlations of EL with g_s_ (r = -0.417, p<0.05) and Tr (r = -0.408, p<0.05). Pn was positively correlated with g_s_ (r = 0.876, p<0.001) and Tr (r = 0.893, p<0.001). The level of correlation between g_s_ and Tr (r = 0.875, p <0.001) was almost the same as that between Pn and g_s_.

**Table 3:** Correlation coefficient (r) among the physiological parameters in 30 switchgrass lines under alkali-salt stress.

Parameters	EL	RWC	Pn	g_s_	Tr
EL	1.000				
RWC	-0.227	1.000			
Pn	-0.34	0.007	1.000		
g_s_	-0.417*	-0.039	0.876***	1.000	
Tr	-0.408*	0.053	0.893***	0.875***	1.000

*, *** represent correlation coefficient (r) was statistically significant at *P* = 0.05, and 0.001, respectively.

We constructed the correlation matrix using the sample mean of five physiological parameters from 30 lines (Table 3). Then the eigenvalues (λ), cumulative percentage of eigenvalues and their eigenvectors (A) were calculated using the correlation matrix. The three eigenvalues (λ_**1**_, λ_**2**_, and λ_**3**_) with the highest numeric values were selected from the five eigenvalues. The sum of these three eigenvalues accounted for 95.5% of the total genetic variance (Table 1). This indicated that the three corresponding eigenvectors (A_1_, A_2_, and A3) were sufficient to represent the five aforementioned physiological indexes for the purpose of evaluating switchgrass tolerance to alkali-salt stress.

As shown in Table 1, the first principal component, eigenvector A_1_, accounted for 59.9% of the variation. The first principal element consisted of high levels of Pn, g_s_ and Tr, and low levels of EL and RWC. Since the factors composing eigenvector A_1_ (Pn, g_s_, and Tr) were related to photosynthesis, the first principal component A_1_ was called the photosynthetic factor. The second principal component, eigenvector A_2_, accounted for 22.5% with the greatest value being RWC. Therefore, we named A_2_ the RWC factor. The third principal component, eigenvector A_3_, accounted for 13.1%, with the greatest value being EL. Therefore, we named A_3_ the EL factor.

The calculated values of the three principal components from 30 switchgrass lines are shown in Table 2. The synthesis scores indicate that six lines, including TEM-SEC, Alamo, TEM-SLC, Kanlow, 70SG001, and T16971, had higher scores, showing they had greater resistance to alkali-salt stress than other lines. In contrast, the last six lines, including AM-314/MS-155, Caddo, BN-8624-67, BN-13645-64,70SG0019, and Blackwell-1, had smaller scores and were considered sensitive to alkali-salt stress. The rest of the lines had medium levels of alkali-salt tolerance.

**Fig 6:**
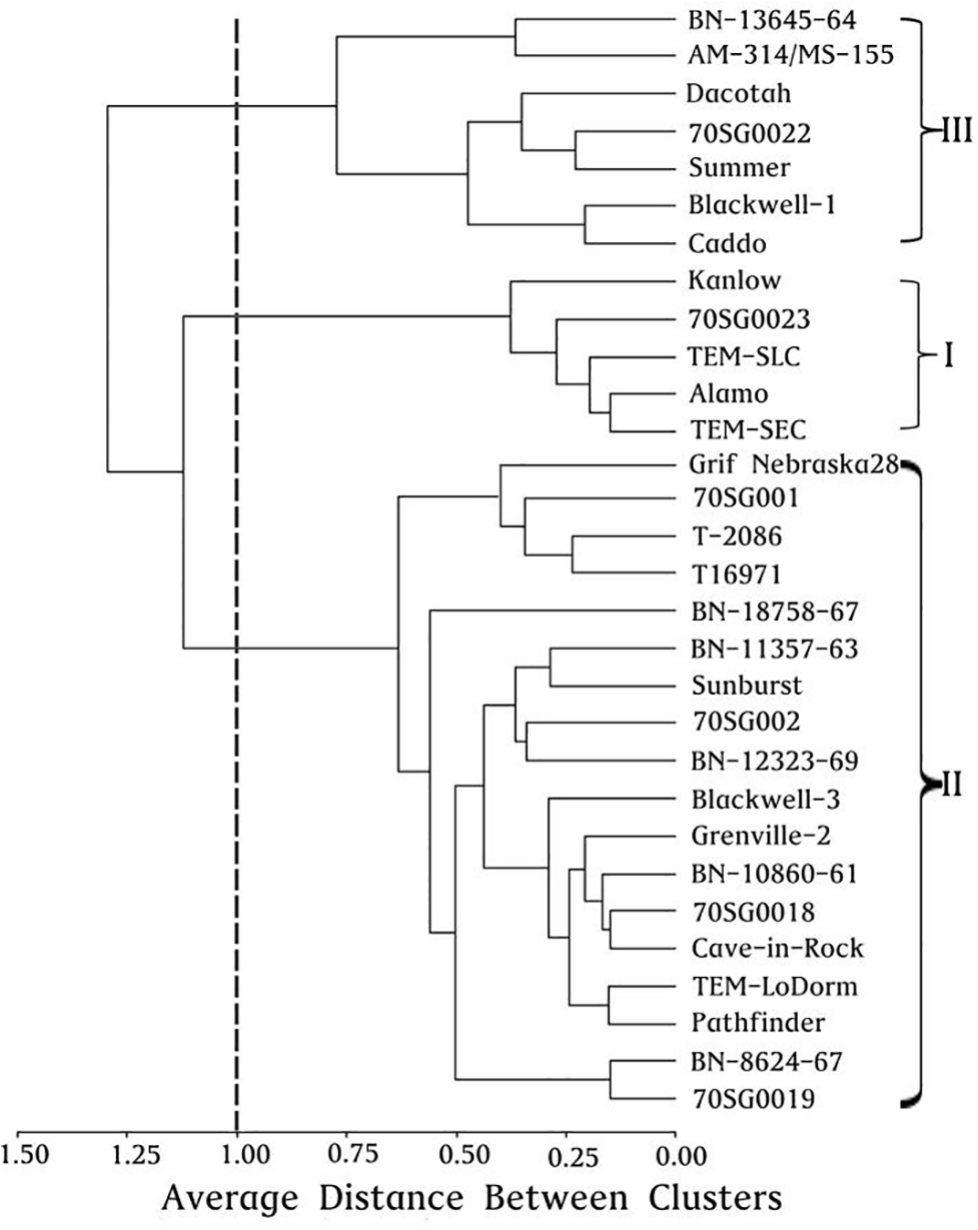
Clusters analysis of five physiological parameters in 30 switchgrass lines.

The physiological parameters in 30 switchgrass lines were used for a cluster analysis. As shown in Fig 6, the experimental lines fell into three categories: Category I (five lines), including TEM-SEC, Kanlow, 70SG0023, Alamo and TEM-SLC, had the lowest level of overall EL and the highest levels of overall Pn, g_s_ and Tr (that is, the highest level of photosynthetic index). These lines accounted for 16.67% of total experiment lines and were considered as alkali-salt stress tolerant lines. Category II (18 lines), including Grif Nebraska 28,70SG0019, Pathfinder, Blackwell-3, BN-8624-67, Cave-in-Rock, T16971, 70SG001, BN-12323-69, 70SG002, BN-18758-67, 70SG0018, TEM-LoDorm, T-2086, Sunburst, BN-11357-63, Grenville-2, and BN-10860-61, had medium levels of all physiological indices. These lines accounted for 60% of total experiment lines. Category III (7 lines), including Caddo, Summer, 70SG0022, AM-314/MS-155, Blackwell-1, BN-13645-64 and Dacotah, had the lowest levels of overall physiological indices and therefore the least tolerant to alkali-salt stress. These lines accounted for 23.3% of total experiment lines and were considered as alkali-salt sensitive lines. Overall, the alkali-salt stress tolerance of three categories was ranked as: Category I > Category II > Category III.

## Discussion

### Selection of Proper Alkali-salt Concentration for Screening Switchgrass Lines

The proper concentration of alkali-salt concentration is critical to evaluate lines for alkali-salt tolerance. In this study, treatment with 150 mM Na^+^ and a pH of 9.5 caused significant physiological responses in the 30 lines. At 6 days after treatment initiation, differences in the physiological parameters were clearly identified among the lines. Fan *et al*. [14] found that the Pn, g_s_, Tr, and Chl content of ‘Alamo’ switchgrass seedlings declined with the NaCl concentration increasing from 50 through 200 mM in a solution culture system, and pointed out that the salt tolerance threshold of switchgrass was 178.6 mM. Du *et al*. [44] noted that salt stress (100–250 mM NaCl) reduced the Chl content and increased EL in switchgrass seedlings. However, Liu et al. [45] found that a salt stress level of 250 mM NaCl was suitable for evaluating the salt tolerance of different switchgrass lines in hydroponic systems. However, no research has been reported on proper concentrations for alkali-salt stress treatment in switchgrass.

Alkali-salt stress exerted the salt stress factors but with the added influence of high-pH stress. Some studies also clearly revealed alkali-salts have a more severe effect on plant growth than neutral salts, such as direct toxicity effects [3, 19, 30, 46–48]. Liu *et al*. [49] found that switchgrass was capable of germinating and surviving well in all treatments under low-alkali pH (pH ≤ 8.3), regardless of the salinity. The three treatment groups designated as C4 (salinity = 110 mM, pH = 10.7), D3 (salinity = 160 mM, pH = 9.5) and D4 (salinity = 160 mM, pH = 10.7) did not show seed germination. This suggests alkali-salts have a more severe effect on plant growth than neutral salts, although switchgrass seeds may not germinate in the soil with pH at 9.5 as reported previously Liu *et al*. [49]. The mature individual plants we used in this study may have greater tolerance to alkali-salt stress relative to young seedlings. Therefore, a pH of 9.5 may be appropriate for screening switchgrass lines for alkali-salt tolerance. In our study a Na^+^ concentration of 150 mM was used, which was slightly lower than the concentration used for aforementioned neutral salt stress. So we suggest that the 150 mM Na^+^ with pH 9.5 may be appropriate for screening switchgrass line for alkali-salt stress tolerance.

### Selection of Physiological Parameters for Screening the Switchgrass Lines

Our results demonstrated that alkali-salt stress (150 mM Na^+^, pH 9.5) caused cell membrane damage (higher EL) and reduced RWC, as well as Pn, g_s_, and Tr, regardless of the line. These results are in agreement with previous studies with sunflower [3], alfalfa [26], wheat [28], *Leymuschinensis* [29], *Puccinelliatenuiflora* [30], *Aneurolepidiumchinense* [31], cotton [33], and tomato plants [50].

Soil salinity and alkalinity interfere with both seed germination and plant growth [49, 51, 52]. Some studies clearly revealed that alkali-salts have more direct toxicity effects on plant growth than neutral salts. Alkali stress can cause the loss of normal physiological functions of the roots and destruction of root cell structure [28]. The high-pH environment surrounding the roots due to alkali stress can directly cause Ca^2+^, Mg^2+^ and H_2_ PO_4_^-^ to precipitate [20], and may inhibit the absorption of ions such as Cl^−^, NO_3_^–^, and H_2_PO_4_^–^ [53], as well as to cause an imbalance of intracellular Na^+^ and K^+^ [32], leading to disruption of ion homeostasis of plant cells [53]. Alkalinity may reduce efficiencies of photon capture and electron transport by PSII and result in Pn decline [54]. This suggests that although the mechanism of alkali-salt stress induced toxicity to plants are more complex than that of neutral salt induced toxicity, the five physiological parameters (Pn, Tr, gs, EL, and RWC) can be used as indicators for assessing the level of alkali-stress tolerance among the switchgrass lines.

The results of our study indicate the 30 lines varied greatly in tolerance to alkali-salt stress based on ASTTI of each parameter. Since Pn was highly correlated with g_s_ and Tr in this study, relative alkali-salt tolerance of the lines was determined mainly based on Pn and ASTTI of Pn. T16971,70SG001, Kanlow, Alamo, TEM-SLC, TEM-SEC, and 70SG0023 were considered alkali-salt tolerant because of higher Pn and the ASTTI, with greater ASTTI values among the 30 lines. In contrast, 70SG0019, BN-8624-67, BN-11357-63, Blackwell-3, AM-314/MS-155, 70SG0018 and Sunburst were alkali-salt sensitive because of lower Pn and ASTTI values in these lines.

Pn and its ASTTI can be considered as the best indicator for alkali-salt tolerance. EL and RWC and their ASTTI can also be used for evaluating alkali-salt stress tolerance. Alkali-salt tolerant lines had relatively lower EL and its ASTTI, whereas alkali-salt tolerant lines had higher EL and its ASTTI values.

### Statistical Model for Evaluating Switchgrass Lines for Alkali-salt Tolerance

Because of weak correlations of Pn with RWC, and EL, the classification of lines in alkali-salt stress tolerance based on Pn may not always match with those according to RWC and EL. Therefore, principal component analysis and cluster analysis were used to further evaluate tolerance to alkali-salt for the 30 lines. The results of this study showed that the information from the five physiological parameters is mainly composed of the three principal components, with cumulative contribution rates accounting for 95.5% of the total genetic variance. This indicates that the three corresponding eigenvectors (A_1_, A_2_, and A_3_) were sufficient to represent the five aforementioned physiological indexes for the purpose of evaluating switchgrass tolerance.

Comprehensive evaluation of the alkali-salt tolerance of switchgrass lines were performed based on principal component analysis. The first principal component consisted of higher levels of Pn, g_s_, and Tr, and negative EL. This indicates that the lines with a greater first principal component (a higher photosynthetic factor and lower EL) were classified as alkali-salt tolerant. This suggests that photosynthetic factors can be used as the primary index for evaluating switchgrass tolerance to alkali-salt stress.

A higher RWC value was found in the second principal component, and greater EL was in the third principal component, therefore, RWC and EL could also be useful parameter in evaluating alkali-salt stress tolerance in switchgrass. A higher level of alkali-salt content in the soil solution may significantly reduce water potential in the root zone. As a result, water in cells may move from inside to outside cell. The water moving out from cell could reduce cell water content (lower RWC), resulting in a shrinking of cell cytoplasm and cell membrane damage (higher EL). A line with higher RWC and lower EL under alkali-salt stress may have less damage and greater resistance to alkali-salt stress than that with lower RWC and higher EL.

The synthesis scores based on to the three principal components (Y_1_, Y_2_, and Y_3_) were used to confirm relative tolerance to alkali-salt stress among the 30 lines. The results indicated the six lines, including TEM-SEC, Alamo, TEM-SLC, Kanlow, 70SG001, and T16971, had higher synthesis scores and can be considered as alkali-salt tolerant. In contrast, the last six lines AM-314/MS-155, Caddo, BN-8624-67, BN-13645-64, 70SG0019, and Blackwell-1had lower synthesis scores and were sensitive to alkali-salt stress. Li et al [42] indicated that the synthesis score is suitable for evaluating quality traits in 11 millet cultivars. This suggests that the synthesis score derived from eigenvectors may be a useful parameter for evaluating alkali-salt tolerance in switchgrass.

In this study, the 30 switchgrass lines fell into three categories based on clustering analysis. Among them, Category I, including five lines (TEM-SEC, Kanlow, 70SG0023, Alamo and TEM-SLC) had the lowest level of overall EL and highest overall Pn, g_s_ and Tr (that is, the greatest photosynthetic index). These lines accounted for 16.7% of total experimental lines and were the most tolerant to alkali-salt stress. There were four lines (TEM-SEC, Alamo, TEM-SLC, and Kanlow) in Category I which coincided with four of previous six lines determined by principal component analysis. Category III included seven lines (Caddo, Summer, 70SG0022, AM-314/MS-155, Blackwell-1, BN-13645-64 and Dacotah) and had the lowest overall physiological indices and therefore are the least tolerant to alkali-salt stress. These lines accounted for 23.3% of total experimental lines. There were four lines (AM-314/MS-155, Caddo, BN-13645-64, and Blackwell-1) in Category III coinciding with four of the six lines with lower tolerance to alkali-salt by principal component analysis. Category II, including 18 lines, with medium levels of all physiological indices, accounted for 60.0% of total experimental lines.

The results of this study indicated that tolerance to alkali-salt stress varied widely among the lines. AM-314/MS-155 and Blackwell-1 exhibited initial leaf wilting on day 6, and complete leaf wilting on day 10 of treatment. This is in agreement with the results by principal component analysis and cluster analysis. There were only three lines (TEM-SEC, Alamo and Grif Nebraska 28) which survived the alkali-salt stress treatment for 20 days. Of the three lines, TEM-SEC and Alamo are lowland ecotypes, while Grif Nebraska 28 is an upland ecotype. All top four lines (TEM-SEC, Alamo, TEM-SLC and Kanlow) based on principal component analysis were lowland ecotypes. This suggests that lowland ecotypes may have greater tolerance to alkali-salt stress than upland ecotypes. This may be related to the environment during evolution of lowland ecotypes. Further investigations on mechanisms of differential tolerance to alkali-salt stress between lowland and upland are warranted.

## Conclusion

Alkali-salt concentration at 150 mM and pH 9.5 was appropriate for screening mature switchgrass plant lines for alkali-salt stress tolerance. The 30 switchgrass lines differed significantly in alkali-salt tolerance. Based on the physiological parameters (EL, RWC, Pn, gs, and Tr), principal component analysis, and cluster analysis, as well as the relative ASTTI values, lowland lines TEM-SEC, Alamo, TEM-SLC and Kanlow were classified as alkali-salt tolerant. In contrast, three lowland lines (AM-314/MS-155, BN-13645-64) and two upland lines (Caddo and Blackwell-1) were classified as alkali-salt sensitive lines. The rest of the lines have moderate tolerance to alkali-salt stress. The selected physiological parameters may be suitable for screening alkali-salt stress tolerance in switchgrass. The results of this study also suggest that a statistical model that combines principal component and cluster analysis of the selected physiological parameters is effective for evaluating alkali-salt tolerance in switchgrass.

## References

[1] Peng YL, Gao ZW, Gao Y, Liu GF, Sheng LX. Eco-physiological characteristics of alfalfa seedlings in response to various mixed salt alkaline stresses. J Integr Plant Biol. 2008; 50: 29–39. 10.1111/j.1744-7909.2007.00607.x 18666949

[2] Flowers TJ. Improving crop salt tolerance. J. Exp. Bot. 2004; 55:307–319. 14718494 10.1093/jxb/erh003

[3] Shi DC, Wang DL. Effects of various salt-alkaline mixed stresses on *Aneurolepidium chinense* (Trin.) Kitag. Plant Soil. 2005; 271: 15–26.

[4] Kim S, Rayburn AL, Voigt T, Parrish A, Lee DK. Salinity effects on germination and plant growth of prairie cordgrass and switchgrass. Bioenergy Res. 2012; 5: 225–235.

[5] Sumner ME, Naidu R. Sodic soils distribution, properties, management, and environmental consequences New York: Oxford University Press; 1998.

[6] Tanji KK. Nature and extent of agricultural salinity In: Tanji KK (ed.) Agricultural salinity assessment and management. pp. 1–8. New York: American Society of Civil Engineers; 1990.

[7] Comis D. Switching to switchgrass makes sense. Agric. Res. 2006; 6:1169–1178.

[8] Srivastava AC, Palanichelvam K, Ma JY, Steele J, Blancaflor EB. Collection and analysis of expressed sequence tags derived from laser capture micro-dissected switchgrass (*Panicum virgatum* L. Alamo) vascular tissues. Bioenergy Res. 2010; 3: 278–294.

[9] Barney JN, DiTomaso JM. Bioclimatic predictions of habitat suitability for the biofuel switchgrass in North America under current and future climate scenarios. Biomass and Bioenergy. 2010; 34: 124–133.

[10] Young HA, Lanzatella CL, Sarath G, Tobias CM. Chloroplast genome variation in upland and lowland switchgrass. PLoS ONE. 2011; 6(8):e23980 10.1371/journal.pome.0023980 21887356 PMC3161095

[11] Lynd LR, Cushman JH, Nelson RJ, Wyman CE. Fuel ethanol from cellulosic biomass. Science. 1991; 251: 1318–1323. 17816186 10.1126/science.251.4999.1318

[12] McLaughlin SB. New switchgrass biofuels research program for the southeast. In: Proc. Annual Automotive Technol. Dev. Contractor's Coordinating Meeting, pp. 111–115. 2–5 November 1992, Dearbon, MI.

[13] Munns R. Comparative physiology of salt and water stress. Plant Cell Environ. 2002; 25:239–250. 11841667 10.1046/j.0016-8025.2001.00808.x

[14] Fan XF, Hou XC, Zuo HT, Wu JY, Duan LS. Effect of marginal land types and transplanting methods on the growth of switchgrass seedlings. Pratacultural Science. 2010; 27:97–102.

[15] McLaughlin SB, Walsh ME. Evaluating environmental consequences of producing herbaceous crops for bioenergy. Biomass and Bioenergy. 1998; 14(4): 317–324.

[16] Alexopoulou E, Sharma N, Papatheohari Y, Christou M, Piscioneri I, Panoutsou C, et al Biomass yields for upland and lowland switchgrass varieties grown in the mediterranean region. Biomass and Bioenergy. 2008; 32:926–933.

[17] Keshwani DR, Cheng JJ. Switchgrass for bioethanol and other value-added applications: A review. Bioresource technology. 2009; 100:1515–1523. 10.1016/j.biortech.2008.09.035 18976902

[18] Gerloff EA, Spukerman E, Proschold T. Effect of external pH on the growth, photosynthesis and photosynthetic electron transport of chlamydomonas acidophila Negoro, isolated from an extremely acidic lake (pH 2.6). Plant Cell Environ. 2005; 28:1218–1229.

[19] Yang CW, Jianaer A, Li CY, Shi DC, Wang DL. Comparison of the effects of salt-stress and alkali-stress on photosynthesis and energy storage of an alkali-resistant halophyte *Chloris virgata* . Photosynthetica. 2008; 46: 273–278.

[20] Shi DC, Zhao KF. Effects of NaCl and Na_2_CO_3_ on growth of *Puccinellia tenuiflora* and on present state of mineral elements in nutrient solution. Acta Pratacu Sin. 1997; 6:51–61.

[21] Netondo GW, John Collins O, Beck E. Sorghum and salinity: II. gas exchange and chlorophyll fluorescence of sorghum under mixed saline-alkaline stress. Crop Sci. 2004; 44:806–811.

[22] Cha-um S, Trakulyingcharoen T, Smitamana P, Kirdmanee C. Salt tolerance in two rice cultivars differing salt tolerant abilities in responses to iso-osmotic stress. Aust. J. Crop Sci. 2009; 3:221–230.

[23] Cha-um S, Kirdmanee C. Salt tolerance screening in six maize (*Zea mays* L.) genotypes using multivariate cluster analysis. Philipp. Agric. Sci. 2010; 93:156–164.

[24] Houimli SIM, Denden M, Mouhandes BD. Effects of 24-epibrassinolide on growth, chlorophyll, electrolyte leakage and proline by pepper plants under NaCl-stress. Eur.Asia J. Bio.Sci. 2010; 4:96–104.

[25] Kanwal H, Ashraf M, Shahbaz M. Assessment of salt tolerance of some newly developed and candidate wheat (*Triticum aestivum* L.) cultivars using gas exchange and chlorophyll fluorescence attributes. Pak. J. Bot. 2011; 43:2693–2699.

[26] Chen W, Feng C, Guo W, Shi DC, Yang CW. Comparative effects of osmotic-, salt- and alkali stress on growth, photosynthesis, and osmotic adjustment of cotton plants. Photosynthetica. 2011;49 (3): 417–425.

[27] Kong-ngern K, Bunnag S, Theerakulpisut P. Proline, hydrogen peroxide, membrane stability and antioxidant enzyme activity as potential indicators for salt tolerance in rice (*Oryza sativa* L.). International J. Bot. 2012; 8:54–65.

[28] Wani AS, Ahmad A, Hayat S, Fariduddin Q. Salt-induced modulation in growth, photosynthesis and antioxidant system in two varieties of *Brassica juncea*. Saudi. J. Biol. Sci. 2013; 20:183–193. 10.1016/j.sjbs.2013.01.006 PMC3730539 23961235

[29] Shi DC, Yin LJ. Strain responses in Na_2_CO_3_ stressed *Leymus chinensis* seedlings and their mathematical analysis. Acta Bot. Sin. 1992; 34: 386–393.

[30] Shi DC, Yin LJ. Difference between salt (NaCl) and alkaline (Na_2_CO_3_) stresses on *Puccinellia tenuiflora* (Griseb.) Scribn et Merr. plants. Acta Bot. Sin. 1993; 35:144–149.

[31] Shi DC, Sheng Y. Effect of various salt-alkaline mixed stress conditions on sunflower seedlings and analysis of their stress factors. Environ. Exp. Bot. 2005; 54: 8–21.

[32] Yang CW, Wang P, Li CY, Shi DC, Wang DL. Comparison of effects of salt and alkali stresses on the growth and photosynthesis of wheat. Photosynthetica. 2008; 46 (1): 107–114.

[33] Mohsenian Y, Roosta HR, Karimi HR, Esmaeilizade M. Investigation of the ameliorating effects of eggplant, datura, orange nightshade, local Iranian tobacco, and field tomato as rootstocks on alkali stress in tomato plants. Photosynthetica 2012; 50 (3): 411–421.

[34] Hoagland DR, Arnon DI. The water-culture method for growing plants with out soil. Circular. Calif. Agric. Exp. Circ. 1950; 247.

[35] Hardin CF, Fu C, Hisano H, Xiao X, Shen H, Stewart CN Jr, et al Standardization of switchgrass sample collection for cell wall and biomass trait analysis. Bioenerg Res. 2013; 6:755–762. 10.1007/s12155-012-9292-1

[36] Chen P, Yan K, Shao H, Zhao S. Physiological mechanisms for high salt tolerance in wild soybean (*Glycine soja*) from Yellow River Delta, China: photosynthesis, osmotic regulation, ionflux and antioxidant capacity. PLoS One. 2013; 8(12):e83227 10.1371/journal.pome.0023580 24349468 PMC3861505

[37] Marcum KB, Anderson SJ, Engelke MC. Salt gland ion secretion: A salinity tolerance mechanism among five zoysiagrass species. Crop Sci.1998; 38:1414–1417.

[38] Weatherley PE. Studies in water relations of cotton plants. I. The field measurement of water deficits in leaves. New Phytol. 1950; 49: 81–97.

[39] Ali Z, Salam A, Azhar FM, Khan IA. Genotypic variation in salinity tolerance among spring and winter wheat (*Triticum aestivum* L.) accessions. S. Afr. J. Bot. 2007; 73:70–75.

[40] Shahzad A, Ahmad M, Iqbal M, Ahmed I, Ali GM. Evaluation of wheat landrace genotypes for salinity tolerance at vegetative stage by using morphological and molecular markers. Genetics and Molecular Res. 2012; 11:679–692. 10.4238/2012.March.19.2 22535404

[41] Tavakkoli E, Fatehi F, Rengasamy P, McDonald GK. A comparison of hydroponic and soil-based screening methods to identify salt tolerance in the field in barley. J. Exp. Bot. 2012;63 (10): 3853–3867. 10.1093/jxb/ers085 22442423 PMC3388819

[42] Li SS, Zhang AX, Wang GR, Zhang XS, Shi ZG, Wang HJ. Correlation and cluster analysis of quality traits in 11 millet cultivar from Shijiazhuang. J. Hunan Agric. Univ. 2012; 6:580–586.

[43] SAS Institute, Inc. 2010 SAS v.9.3. SAS Institute, Inc Cary, NC, USA.

[44] Du F, Chen X, Yang CH, Zhang YW. Effects of NaCl stress on seed germination and seedling growth of different switchgrass materials. Acta Agrestia Sinica. 2011; 19:1018–1024.

[45] Liu YM, Zhang XZ, Miao JM, Huang LK, Frazier T, Zhao BY. Evaluation of Salinity Tolerance and Genetic Diversity of Thirty-Three Switchgrass (*Panicum virgatum*) Lines. Bioenerg. Res. 2014; 7:1329–1342. 10.1007/s12155-014-9466-0

[46] Tang C, Turner NC. The influence of alkalinity and water stress on the stomatal conductance, photosyn6hetic rate and growth of *Lupinus angustifolius* L. and *Lupinus pilosus* Murr. Aust. J Exp.Agric. 1999; 39:457–464.

[47] Rao PS, Mishra B, Gupta SR, Rathore A. Reproductive stage tolerance to salinity and alkalinity stresses in rice genotypes. Plant Breeding. 2008; 127: 256–261.

[48] Li R, Shi F, Fukuda K. Interactive effects of various salt and alkali stresses on growth, organic solutes, and cation accumulation in a halophyte *Spartina alterniflora* (Poaceae). J. Environ. Exp.Bot. 2010; 68: 66–74.

[49] Liu Y, Wang QZ, Zhang YW, Cui J, Chen G, Xie B, et al Synergistic and antagonistic effects of salinity and pH on germination in switchgrass (*Panicum virgatum* L.). PLoS ONE. 2014; 9 (1):e85282 10.1371/journal.pone.0083227 24454834 PMC3891870

[50] Peng YL, Gao ZW, Gao Y, Liu GF, Sheng LX, Wang DL. Eco-physiological Characteristics of alfalfa seedlings in response to various mixed salt-alkaline stresses. J. Integr.Plant Biol. 2008; 50 (1): 29–39. 10.1111/j.1744-7909.2007.00607.x 18666949

[51] Thomson JA, Alonso-Amelot ME. Clarification of the taxonomic status and relationships of *Pteridium caudatum* (Dennstaedtiaceae) in central and south America. Bot. J. Linnean Soc. 2002; 140: 237–248.

[52] Guo R, Zhou J, Hao WP, Gong DZ, Zhong XL. Germination, growth, photosynthesis and ionic balance in *Setariaviridis* seedlings subjected to saline and alkaline stress. Canadian J. Plant Sci. 2011; 91: 1077–1088.

[53] Yang CW, Chong JN, Li CY, Kim CM, Shi DC. Osmotic adjustment and ion balance traits of an alkali resistant halophyte Kochia sieversiana during adaptation to salt and alkali conditions. Plant Soil. 2007; 294: 263–276.

[54] Wu ZH, Yang CW, Yang MY. Photosynthesis, photosystem II efficiency, amino acid metabolism and ion distribution in rice (*Oryza sativa* L.) in response to alkaline stress. Photosynthetica.2014; 52 (1): 157–160. 10.1007/s11099-014-0002-4

